# Ginkgolic Acid C 17:1, Derived from *Ginkgo biloba* Leaves, Suppresses Constitutive and Inducible STAT3 Activation through Induction of PTEN and SHP-1 Tyrosine Phosphatase

**DOI:** 10.3390/molecules22020276

**Published:** 2017-02-13

**Authors:** Seung Ho Baek, Jong Hyun Lee, Chulwon Kim, Jeong-Hyeon Ko, Seung-Hee Ryu, Seok-Geun Lee, Woong Mo Yang, Jae-Young Um, Arunachalam Chinnathambi, Sulaiman Ali Alharbi, Gautam Sethi, Kwang Seok Ahn

**Affiliations:** 1 College of Korean Medicine, Kyung Hee University, 24 Kyungheedae-ro, Dongdaemun-gu, Seoul 02447, Korea; baeksh@woosuk.ac.kr (S.H.B.); 88milkyway@hanmail.net (J.H.L.); sunny10526@nate.com (C.K.); gokjh1647@gmail.com (J.-H.K.); seokgeun@khu.ac.kr (S.-G.L.); wmyang@khu.ac.kr (W.M.Y.); jyum@khu.ac.kr (J.-Y.U.); 2 College of Korean Medicine, Woosuk University, 46 Eoeun-ro, Wansan-gu, Jeonju-si, Jeollabuk-do 54987, Korea; 3 Department of Radiation Oncology, University of Ulsan College of Medicine, Asan Medical Center, Seoul 05505, Korea; rshgood@hanmail.net; 4 Department of Botany and Microbiology, College of Science, King Saud University, Riyadh 11451, Saudi Arabia; dr.arunmicro@gmail.com (A.C.); sharbi@ksu.edu.sa (S.A.A.); 5 School of Biomedical Sciences, Curtin Health Innovation Research Institute, Curtin University, Perth, WA 6009, Australia; 6 Department of Pharmacology, Yong Loo Lin School of Medicine, National University of Singapore, Singapore 117600, Singapore

**Keywords:** ginkgolic acid C 17:1, STAT3, PTEN, SHP-1, apoptosis

## Abstract

Ginkgolic acid C 17:1 (GAC 17:1) extracted from *Ginkgo biloba* leaves, has been previously reported to exhibit diverse antitumor effect(s) through modulation of several molecular targets in tumor cells, however the detailed mechanism(s) of its actions still remains to be elucidated. Signal transducer and activator of transcription 3 (STAT3) is an oncogenic transcription factor that regulates various critical functions involved in progression of diverse hematological malignancies, including multiple myeloma, therefore attenuating STAT3 activation may have a potential in cancer therapy. We determined the anti-tumor mechanism of GAC 17:1 with respect to its effect on STAT3 signaling pathway in multiple myeloma cell lines. We found that GAC 17:1 can inhibit constitutive activation of STAT3 through the abrogation of upstream JAK2, Src but not of JAK1 kinases in U266 cells and also found that GAC can suppress IL-6-induced STAT3 phosphorylation in MM.1S cells. Treatment of protein tyrosine phosphatase (PTP) inhibitor blocked suppression of STAT3 phosphorylation by GAC 17:1, thereby indicating a critical role for a PTP. We also demonstrate that GAC 17:1 can induce the substantial expression of PTEN and SHP-1 at both protein and mRNA level. Further, deletion of *PTEN* and *SHP-1* genes by siRNA can repress the induction of PTEN and SHP-1, as well as abolished the inhibitory effect of drug on STAT3 phosphorylation. GAC 17:1 down-regulated the expression of STAT3 regulated gene products and induced apoptosis of tumor cells. Overall, GAC 17:1 was found to abrogate STAT3 signaling pathway and thus exert its anticancer effects against multiple myeloma cells.

## 1. Introduction

Multiple myeloma is the second most common hematological malignancy [1], characterized by a multifocal proliferation of clonal, long-lived plasma cells within the bone marrow and associated with the skeletal destruction, serum monoclonal gammopathy, immune suppression, and end-organ sequelae [2]. Two-thirds of newly diagnosed patients with multiple myeloma are over 65 years and/or physically unfit [3]. Therefore, novel treatment modalities that can prolong survival and improve quality of life while maintaining good tolerability profiles in patients are required. Chemotherapeutic drugs, such as thalidomide, bortezomib, lenalidomide are the most commonly used treatment options; however multiple myeloma still remains incurable because of toxicity and poor response rates to these drugs. Thus, more effective therapeutic agents are urgently needed for its therapy.

Prior studies have reported that signal transducer and activator of transcription 3 (STAT3) is an oncogenic transcription factor that can regulate many critical functions including cell proliferation, differentiation and apoptosis in tumor cells [4,5]. Among STAT family members, STAT3 in particular is often persistently activated in various human cancer cell lines such as multiple myeloma [6], leukemia, lymphoma, and solid tumors [7]. The expression of various gene products required for tumorigenesis (e.g., Survivin, Bcl-xl, Bcl-2), proliferation (e.g., Cyclin D1), invasion (MMP-9), and angiogenesis (e.g., VEGF) can be elicited by phosphorylation of STAT3 [7,8]. STAT3 activation has also been implicated in decreased survival in patients with multiple myeloma [9]. Phosphorylation at tyrosine residue (Tyr705) is critical for STAT3 activation, which contributes to dimerization, leading to nuclear translocation, DNA binding, and subsequent gene transcription. This phosphorylation is mediated through the activation of intracellular tyrosine kinases called janus-like kinase (JAK). JAK1, JAK2, JAK3, and TYK2 have been linked to the activation of STAT3 [10,11]. STAT3 has also been proposed to be regulated by c-Src [12]. Whereas these kinases positively regulate the STAT3 pathway, various protein tyrosine phosphatases (PTPs), such as PTEN [13] and SHP-1 [14] have been identified to negatively regulate STAT3 activation. Also, various prior reports indicate that pharmacological agents that can abolish STAT3 activation may have a great potential in the prevention and therapy of cancer [15].

Since approximately 74.8% (131/175) of all anti-cancer drugs approved either have been isolated from natural sources or mimic them in one form or another (1981–2010) [16,17], agents derived from natural sources could be one potential source of STAT3 inhibitors for targeting the proliferation and survival of tumor cells [18,19,20,21]. One potentially useful source of diverse natural compounds is the plant *Ginkgo biloba* whose leaves contain various glycosides and terpenoids that can exert diverse pharmacological activities [22,23,24,25], such as cardiotonic effect for the prevention and treatment of cardiovascular disease [26,27]. Among various compounds, ginkgolic acid C 17:1 and ginkgolic acid C 15:1 are the most abundant ones isolated from this medicinal plant [28]. It has been demonstrated that ginkgolic acid can inhibit the proliferation of various tumor cell types, including pancreatic [29], breast, lung, and leukemia [30], and also induce substantial apoptosis [29,31,32]. Ginkgolic acid exhibits its antitumor effects through the modulation of several oncogenic targets including attenuation of pathways involved in lipogenesis [29], cell cycle arrest and decrease of the Bcl-2/Bax ratio [31]. Especially, it has been suggested that ginkgolic acid can cause inhibition of SUMOylation that controls diverse cellular functions and its de-regulation may lead to both cancer and neurodegenerative diseases [33,34]. These reports suggest that ginkgolic acid could be an attractive candidate agent for cancer treatment, although the detailed mechanism(s) through which ginkgolic acid exhibits its pharmacological effects still remains to be elucidated.

In this study, we specifically investigated whether ginkgolic acid can suppress the STAT3 pathway in multiple myeloma cells and the underlying molecular mechanism(s) involved. We found for the first time that GAC indeed inhibits both constitutive and inducible STAT3 activation leading to the suppression of cell proliferation and down-regulation of various gene products that prevent apoptosis and promote inflammation and metastasis in tumor cells.

## 2. Results

### 2.1. GAC 17:1 Selectively Exerted Cytotoxic Effects against Multiple Myeloma Cells

**Figure 1:**
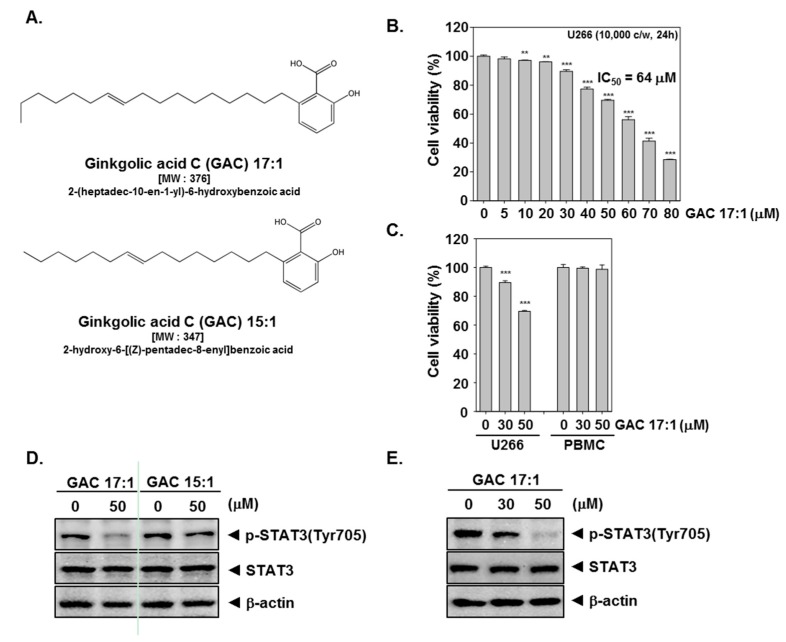

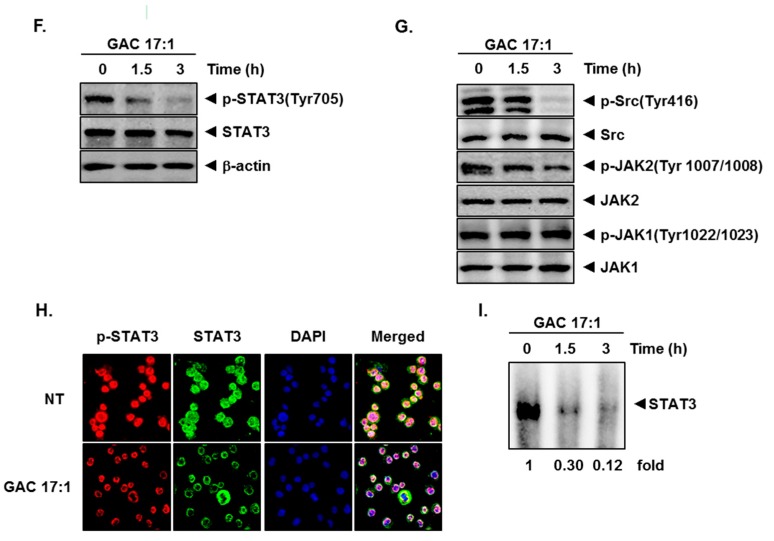
Inhibitory effects of GAC 17:1 on constitutively activated STAT3 in U266 cells. (**A**) The chemical structure of GAC 17:1 and GAC 15:1; (**B**,**C**) U266 cells and PBMCs (1 × 10^4^ cells/well) were treated with various concentrations of GAC 17:1 for 24 h and cell viability was determined by MTT assay, ** *p* < 0.01 and *** *p* < 0.001 indicates significant differences from the control group; (**D**) U266 cells (5 × 10^5^ cells/well) were treated with GAC 17:1 (50 μM) or GAC 15:1 (50 μM) for 3 h. Whole-cell extracts were prepared then equal amount of lysates were subjected to western blot analysis for p-STAT3 (Tyr705), STAT3, and β-actin; (**E**) U266 cells (5 × 10^5^ cells/well) were incubated with GAC 17:1 (30 or 50 μM) for 3 h. Thereafter, equal amounts of lysates were analyzed by western blot analysis using antibodies against for p-STAT3 (Tyr705), STAT3, and β-actin; (**F**,**G**) U266 cells (5 × 10^5^ cells/well) were treated with GAC 17:1 (50 μM) for the indicated time. Lysates from the cells were analyzed using western blot analysis for p-STAT3 (Tyr705), STAT3, p-JAK1 (Tyr1022/1023), JAK1, p-Src (Tyr416), and Src; (**H**) After 3 h of GAC 17:1 (50 μM) treatment, the cells were fixed and permeabilized. STAT3 (green) and p-STAT3 (red) were immunostained with rabbit anti-Stat3 and goat anti-p-STAT3 respectively followed by FITC-conjugated secondary antibodies, then nucleus (blue) was stained with DAPI. The fourth panels show the merged images of the first, second and third panels; (**I**) U266 cells (5 × 10^5^ cells/well) were incubated with the GAC 17:1 (50 μM) for 1.5 or 3 h. Nuclear extracts were prepared for detection of STAT3 binding activities and testing was performed by EMSA.

The structure of ginkgolic acid C 17:1 (GAC 17:1) and ginkgolic acid C 15:1 (GAC 15:1) are shown in Figure 1A. The cytotoxic effects of GAC 17:1 against U266 and PBMCs were evaluated by MTT assay. We found that GAC 17:1 treatment reduced the viability of U266 cells in a concentration dependent manner with IC_50_ value of approximately 64 μM (Figure 1B) but not PBMCs (Figure 1C).

### 2.2. GAC 17:1 Suppressed Constitutive STAT3 Phosphorylation in U266 Cells

We determined effect of GAC 17:1 using multiple myeloma U266 cells in which STAT3 has been found to be constitutively activated. First, the effect of GAC 17:1 and GAC 15:1 was examined by western blotting in U266 cells. As shown in Figure 1D GAC 17:1 can inhibit the constitutively active STAT3 in U266 cells, while GAC 15:1 was not able to suppress the activation of STAT3; Next, U266 cells were treated with the GAC 17:1 (30 or 50 μM) for 3 h or incubated with GAC 17:1 (50 μM) for 1.5 or 3 h. Figure 1E,F show that GAC 17:1 substantially suppresses phosphorylation of STAT3 in a dose- and time-dependent manner. However, STAT3 expression was not affected by GAC 17:1. Thus, we proceeded to determine the in depth molecular mechanisms of GAC 17:1-induced inhibition of STAT3 signaling pathway in U266 cells.

### 2.3. GAC 17:1 Attenuated Phosphorylation of Upstream Kinases in U266 Cells

Because STAT3 activation is mediated through Src and JAK families [35,36], we set out to determine whether GAC 17:1 could modulate constitutive phosphorylation of Src, JAK2, and JAK1 kinases in U266 cells. As shown in Figure 1G, substantial decrease of Src and JAK2 activation could be noted in a time dependent manner following GAC 17:1 (50 μM) treatment.

### 2.4. GAC 17:1 Repressed Nuclear Translocation of STAT3 in U266 Cells

Tyrosine phosphorylation causes dimerization of STAT3 and nuclear translocation [9,18], hence we next examined whether GAC 17:1 can also affect nuclear translocation of STAT3 in U266 cells by immunocytochemistry. Figure 1H shows that GAC 17:1 abolishes STAT3 translocation from the cytoplasm to the nucleus in U266 cells.

### 2.5. GAC 17:1 Inhibited Binding of STAT3 to the DNA

Once STAT3 is translocated to the nucleus, it binds to the DNA and regulates STAT3-dependent gene expression [37]. We next examined whether GAC 17:1 inhibits nuclear location of STAT3 by EMSA. Nuclear extracts prepared from U266 cells show that GAC 17:1 clearly inhibited STAT3-DNA binding activity in a time-dependent manner (Figure 1I).

### 2.6. GAC 17:1 Decreased IL-6-Induced Phosphorylation of STAT3 in U266 Cells

**Figure 2:**
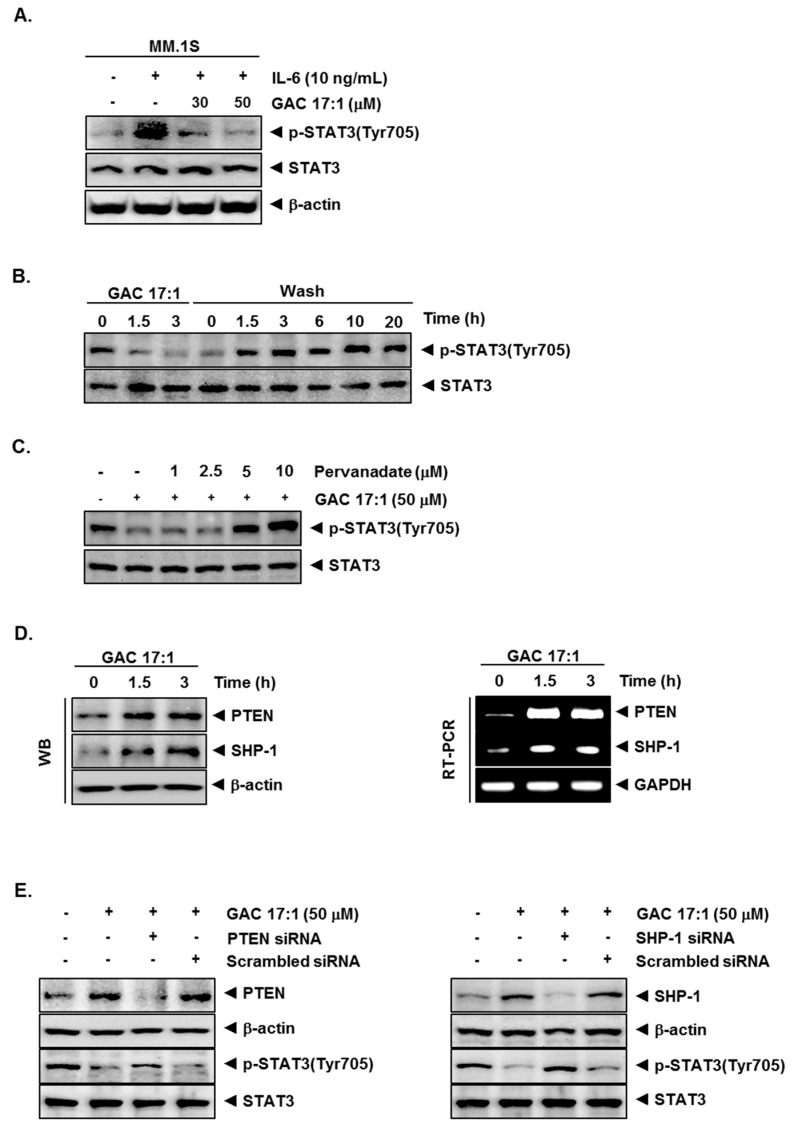
Inhibition of IL-6 inducible STAT3 activation and the induction of PTEN and SHP-1 proteins by GAC 17:1 in multiple myeloma cells. (**A**) MM1.S cells (5 × 10^5^ cells/well) were pretreated with GAC 17:1 (50 μM) for 3 h and then stimulated with IL-6 (10 ng/mL) for 15 min to induce activation of STAT3. Whole-cell extracts were prepared and immunoblotted with antibodies for phospho-STAT3 (Tyr705) and STAT3; (**B**) U266 cells (5 × 10^5^ cells/well) were treated with GAC 17:1 (50 μM) for the indicated time and washed with PBS to remove GAC 17:1 before resuspension in fresh medium. Cells were removed at indicated times and lysed to prepare the whole-cell extract, then analyzed using Western blot analysis for p-STAT3 (Tyr705) and STAT3; (**C**) U266 cells (5 × 10^5^ cells/well) were treated with 50 μM of GAC 17:1 for 3 h following pretreatment of the indicated concentrations of pervanadate for 30 min. Whole-cell extracts were prepared and immunoblotted with antibodies for p-STAT3 (Tyr705) and STAT3; (**D**) U266 cells (5 × 10^5^ cells/well) were treated with GAC 17:1 (50 μM) for 1.5 or 3 h. Whole-cell extracts were prepared, equal amounts of lysates were analyzed by western blot analysis using antibodies against PTEN and SHP-1. And total RNA was extracted and examined for expression of PTEN and SHP-1 by RT-PCR; (**E**) U266 cells were transfected with scrambled or PTEN or SHP-1-specific siRNA (50 nM). After 24 h, cells were treated with GAC 17:1 (50 μM) for 3 h and the whole-cell extracts were subjected to western blot analysis for PTEN, SHP-1, p-STAT3 (Tyr705), and STAT3. β-Actin was used as a loading control.

IL-6, a growth factor for multiple myeloma cells, is overexpressed in various cancers and is a potent inducer of STAT3. Whether GAC 17:1 can also abrogate IL-6-induced STAT3 phosphorylation in MM.1S cells was determined. We observed that IL-6 induced phosphorylation of STAT3 as early as 15 min, and pretreatment of GAC 17:1 inhibited IL-6-induced STAT3 phosphorylation (Figure 2A).

### 2.7. Inhibition of STAT3 Phosphorylation by GAC 17:1 Is Reversible

Whether GAC 17:1-induced inhibition of STAT3 activation was reversible was also investigated. Figure 2B shows that removal of the compound can reverse the GAC 17:1-induced inhibition of STAT3 phosphorylation (Figure 2A). The level of total STAT3 protein was not affected under these conditions.

### 2.8. Tyrosine Phosphatase Inhibitor Blocked the Suppression of STAT3 Phosphorylation by GAC 17:1

**Figure 3:**
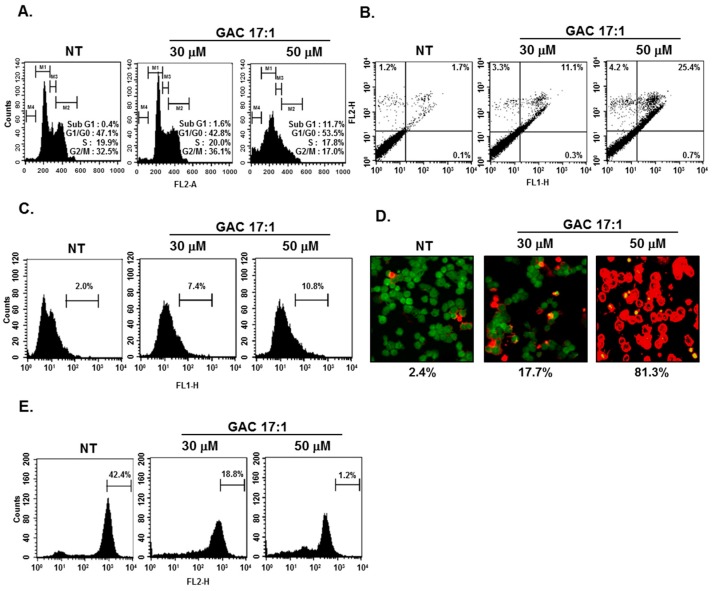
Apoptotic effects of GAC 17:1 in U266 cells. (**A**) U266 cells were treated with GAC 17:1 (50 μM) for 24 h. The cells were harvested, washed with a cold PBS buffer and digested with RNase A. Cellular DNA was stained with propidium Iodide and a flow cytometric analysis was done to determine the cell cycle distribution; (**B**) U266 cells were treated with GAC 17:1 (50 μM) for 24 h and the cells were incubated with Annexin-V/FITC and propidium iodide, then analyzed by a flow cytometer; (**C**) Apoptosis in U266 cells was detected by TUNEL assay. After treatment, the cells were stained with a TUNEL assay reagent and then analyzed under a flow cytometer; (**D**) After 24 h of GAC 17:1 (50 μM) treatment, cells were stained with a Live/Dead assay reagent for 30 min and then analyzed under a confocal microscope; (**E**) U266 cells were treated with GAC 17:1 (50 μM) for 24 h. The cells were washed with a PBS and treated with 5 μM of TMRE (tetramethylrhodamine, ethyl ester) and then analyzed by a flow cytometer to detect MMP activity.

Because protein tyrosine phosphatases (PTPs) have been implicated in STAT3 activation [38], therefore, we next determined whether GAC 17:1-induced inhibition of STAT3 phosphorylation could be due to activation of a PTP. Treatment of U266 cells with the broad-acting tyrosine phosphatase inhibitor sodium pervanadate reversed the GAC 17:1-induced suppression of STAT3 phosphorylation (Figure 3E). This result indicated that PTPs may play an important role in inhibition of STAT3 phosphorylation.

### 2.9. GAC 17:1 Induced the Expression of PTEN and SHP-1 in Protein and mRNA Level

Since PTEN and SHP-1 have been reported to regulate STAT3 signaling pathway [39,40], whether GAC 17:1 has the potential to modulate the expression of PTEN and SHP-1 proteins was investigated. Our result shows that GAC 17:1 can induce the expression of PTEN and SHP-1 in a time-dependent manner. (Figure 2D upper panels). We also examined the effect of GAC 17:1 on the expression of PTEN and SHP-1 in mRNA level by RT-PCR. We found that GAC 17:1 clearly up-regulated the expression of both PTEN and SHP-1 mRNA in a time-dependent manner (Figure 2D lower panels). GAPDH was used as an internal control to show equal RNA loading.

### 2.10. Silencing of SHP-1 and PTEN Reversed the Effect of GAC 17:1 on STAT3 Activation

Whether the inhibition of PTEN and SHP-1 by siRNA can attenuate the effect of GAC 17:1 on STAT3 activation was determined. As shown in Figure 2E, the deletion of PTEN and SHP-1 substantially suppressed the GAC 17:1-induced inhibition of STAT3 phosphorylation. These results indicated that both PTEN and SHP-1 may play a critical role in the suppression of STAT3 activation.

### 2.11. GAC 17:1 Caused the Accumulation of the Cells in the Sub-G1 Phase of the Cell Cycle, and Increased Annexin V Positive Cells

Because suppression of aberrantly activated STAT3 could lead to apoptosis [41,42], we determined whether GAC 17:1 could also induce apoptosis using cell cycle analysis. After 24 h of GAC 17:1 (30 or 50 μM) treatment, the sub-G1 DNA contents were substantially increased in a dose-dependent manner. (Figure 3A). Next, U266 cells treated with GAC 17:1 (30 or 50 μM) for 24 h were incubated with Annexin-V/FITC and PI and then analyzed by a flow cytometer. Figure 3B shows that the Annexin V positive cells were clearly increased in GAC 17:1-treated cells in a dose-dependent manner.

### 2.12. GAC 17:1 Elicited Apoptosis and Caused Loss of Mitochondrial Membrane Potential in U266 Cells

Next TUNEL assay and Live/Dead assay were performed to examine the apoptotic effect of GAC 17:1. For this, U266 cells were exposed to 30 or 50 μM of GAC 17:1 for 24 h. Our results indicated that GAC 17:1 can significantly induce apoptosis in U266 cells (Figure 3C,D). In addition, as the mitochondrial membrane potential decreases during apoptosis [43], flow cytometric analysis was performed to determine the possible changes in mitochondrial membrane potential upon drug exposure. The percentage of cells with higher fluorescent intensities was clearly decreased in the cells treated with GAC 17:1 (30 or 50 μM) for 24 h (Figure 3E).

### 2.13. GAC 17:1 Suppressed the Proliferation of Multiple Myeloma Cells

**Figure 4:**
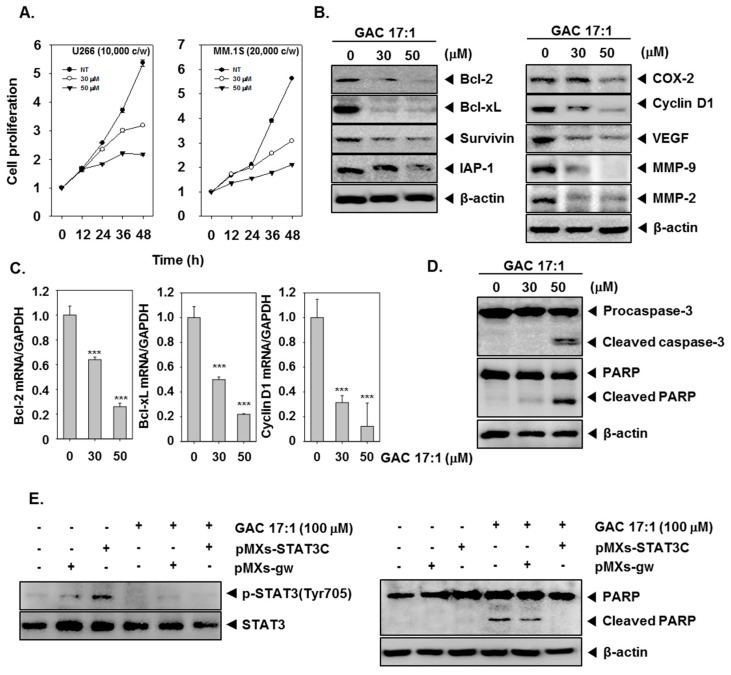
Suppression of STAT3 regulated gene products by GAC 17:1 in multiple myeloma cells. (**A**) U266 and MM.1S cells (1 or 2 × 10^4^ cells/well) were incubated with the GAC 17:1 (30 or 50 μM) for the indicated time intervals. MTT assay was performed to investigate the effect of GAC 17:1 on cell proliferation; (**B**–**D**) U266 cells (3 × 10^5^ cells/well) were incubated with the GAC 17:1 (30 or 50 μM) for 24 h. Lysates from the cells were analyzed using western blotting against Bcl-2, Bcl-xL, Survivin, IAP-1, COX-2, Cyclin D1, VEGF, MMP-9,MMP-2, Caspase-3, and PARP. In addition, Total RNA was isolated, and analyzed by real time PCR for Bcl-2, Bcl-xL, and Cyclin D1. Data represents means ± S.D., *** indicates *p* < 0.001; (**E**) MEF cells were transfected pMXs-STAT3C or pMXs-STAT3gw and incubated for 24 h. The cells were treated with GAC 17:1 (100 μM) for 3 h (left panels) or 24 h (right panels). The lysates from the cells were analyzed by Western blotting using antibodies against p-STAT3 (Tyr705) and STAT3 (left panels) or PARP and β-actin (right panels) respectively.

As GAC 17:1 inhibited phosphorylation of STAT3 which plays an important role in tumor cell proliferation [44], we next examined whether cell proliferation of U266 and MM1.S cells can also be affected by GAC 17:1. The cells were treated with GAC 17:1 (30 or 50 μM) for the indicated time intervals and then MTT assay was performed to analyze cell proliferation. Figure 4A demonstrates that GAC 17:1 significantly repressed cell proliferation in both U266 and MM.1S cells in a dose- and time-dependent manner (Figure 3A).

### 2.14. GAC 17:1 Down-Regulated the Expression of STAT3-Regulated Gene Products

Since constitutive activation of STAT3 regulates the expression of various gene products involved in proliferation, anti-apoptosis, invasion, and angiogenesis, thus we determined whether GAC 17:1 could suppress STAT3-regulated gene expression. The results show that GAC 17:1 treatment down-regulated the expression of anti-apoptotic proteins, such as Bcl-2, Bcl-xL, Survivin and IAP-1 and expression of COX-2, Cyclin D1, VEGF, MMP-9, and MMP-2 involved in cell proliferation, angiogenesis, and metastasis in a dose-dependent manner (Figure 4B). GAC 17:1 (30 or 50 μM) treatment also reduced mRNA expression of Bcl-2, Bcl-xL, and Cyclin D1 in a similar manner (Figure 4C).

### 2.15. GAC 17:1 Induced the Cleavage of Caspase-3 and PARP

Next, we examined the effect of GAC 17:1 on the activation of caspase-3 and PARP. As shown in Figure 4D, GAC 17:1 treatment activated caspase-3 in a dose-dependent manner. We also found that GAC 17:1 caused PARP cleavage in a similar manner (Figure 4D). These results suggest that GAC 17:1 obviously induces caspase-3-dependent apoptosis in U266 cells.

### 2.16. GAC 17:1 Inhibited pMXs-STAT3C-Induced Phosphorylation of STAT3 in MEF Cells

We further investigated the inhibitory effect of GAC 17:1 on inducible STAT3 activation. For this, MEF cells were transfected with pMXs-STAT3C and incubated with 100 μM of GAC 17:1 for 3 h, and then examined for phosphorylated STAT3 by western blot analysis. As shown in Figure 4E, transfection with pMXs-STAT3C clearly caused an increased phosphorylation of STAT3 and GAC 17:1 (100 μM) significantly inhibited pMXs-STAT3C-induced activation of STAT3.

### 2.17. Activation of STAT3 Abolished the Apoptotic Effect of GAC 17:1 in MEF Cells

Next, we examined whether GAC 17:1 can induce apoptosis in MEF cells and activation of STAT3 can mediate the apoptotic effect of GAC 17:1. As shown in Figure 4F, GAC 17:1 (100 μM) caused an increased apoptosis in MEF cells and the induction of apoptosis by GAC 17:1 was abrogated upon transfection with pMXs-STAT3C. These results indicated that anti-apoptotic effects of STAT3 signaling pathway can block the induction of apoptosis by GAC 17:1.

## 3. Discussion

Persistent STAT3 activation has been linked with several chronic diseases [45,46,47], including various cancers [48,49,50,51]. Given the pivotal role of STAT3 in multiple myeloma initiation and progression, the aim of our study was to investigate whether GAC 17:1 can exert its anti-tumor activity through the attenuation of the STAT3 signaling pathway in multiple myeloma cells. We found that GAC 17:1 inhibited both constitutive and IL-6-inducible STAT3 activation in multiple myeloma cells along with the abrogation of c-Src and JAK2 activation and the induction of PTEN and SHP-1 proteins. This inhibition was linked to the down-regulation of numerous oncogenic proteins leading to the inhibition of proliferation, induction of apoptosis, and significant suppression of the growth of multiple myeloma cells. Additionally, it has been previously reported that GAC 17:1 exhibits minimal toxicity towards normal cells [29,31] and we also observed that the drug was comparatively less toxic towards normal cells. Overall, these results demonstrate that GAC 17:1 could be an effective anticancer agent that is predominantly safe and can act as a novel blocker of STAT3 activation in multiple myeloma.

We demonstrated for the first time that GAC 17:1 can inhibit activation of STAT3 in U266 cells and also found that GAC 17:1 suppressed IL-6-induced STAT3 activation in multiple myeloma and MEF cells. We noted that GAC 17:1 can indeed inhibit STAT3 activation at Tyr705 as observed by western blotting, its DNA binding as determined by EMSA, and its nuclear translocation as analyzed by immunohistochemistry in U266 cells. Since STAT3 is constitutively active in multiple myeloma cell lines and contributes to chemoresistance [52,53], these findings may have a great significance in the therapy of multiple myeloma.

The underlying molecular mechanism(s) through which GAC 17:1 can inhibit STAT3 activation was also investigated in detail. STAT3 can directly interact with JAK1 and JAK2 kinases as scaffold, and this interaction can lead to STAT3 phosphorylation at Tyr 705 [17]. We showed that GAC 17:1 treatment causes substantial abrogation of JAK2 phosphorylation level in a time-dependent manner that may be directly implicated in its STAT3 inhibitory effects in multiple myeloma cells. Besides causing attenuation of JAK2 activation, GAC 17:1 can also abrogate c-Src phosphorylation involved in STAT3 activation in U266 cells. Therefore, we can conclude that GAC 17:1 may down-regulate STAT3 activation by repressing the phosphorylation of both Src and JAK2 proteins.

We also found evidence that GAC-induced inhibition of STAT3 activation may be linked with the induction of PTPs. Previous studies have reported that numerous PTPs have been closely associated with STAT3 phosphorylation [51]. In this study, we observed that GAC 17:1 down-regulated STAT3 activation through the induction of both PTEN and SHP-1 proteins in U266 cells, which related to its inhibitory effect on STAT3 phosphorylation. Deletion of the *PTEN* gene by siRNA abrogated the effect of GAC 17:1 on STAT3 phosphorylation, thereby clearly indicating that the PTP plays an important role in down-regulation of STAT3 by GAC 17:1. Moreover, silencing of SHP-1 gene by transfection also reversed the GAC 17:1-induced inhibition of STAT3. Since it has been previously suggested that various pharmacological agents may exhibit their effects through the up-regulation of PTEN [41,42,54] or SHP-1 [55,56,57] expression, GAC 17:1 was found to simultaneously induce both PTEN and SHP-1 expression and thus can also form a basis of novel strategy to modulate STAT3 activation in cancer cells.

Because constitutive STAT3 activation can induce specific target genes that may stimulate cell proliferation, prevent apoptosis and promote angiogenesis [52], we examined whether GAC 17:1 can suppress the expression of STAT3-regulated gene products, overexpressed in multiple myeloma cells [53], including proliferative gene product cyclin D1, COX-2, the angiogenic protein VEGF, and anti-apoptotic gene products, such as IAP, survivin, Bcl-2, and Bcl-xL. Therefore, the effect of GAC 17:1 on the expression of various STAT3 regulated oncogenic genes or gene products were analyzed by Western blotting, RT-PCR, and real-time PCR. We demonstrate that GAC 17:1 suppressed the expression of various STAT3-regulated proteins, including Bcl-2, Bcl-xL, survivin, IAP-1, COX-2, Cyclin D1, MMP-9, MMP-2, and VEGF. The down-regulation of bcl-2 by GAC 17:1 that we found is in agreement with previous reports [31]. Previous studies have already shown that anti-apoptotic proteins, Bcl-2, Bcl-xL and survivin are targets of STAT3 and overexpression of these proteins promote cell survival, enhance cellular resistance for chemo radiation and inhibit apoptosis [55,56]. Therefore, we propose that GAC 17:1 may induce apoptosis by inhibition of STAT3 activation which can lead to the reduction of anti-apoptotic proteins. Since VEGF expression can also be modulated by STAT3, GAC 17:1 may also exert anti-angiogenic effect by modulating VEGF expression [55].

Recent studies have suggested that constitutively-activated STAT signaling can directly contribute to oncogenesis [58] and increase Bcl-xL expression which can inhibit induction of apoptosis [6]. The reduced expression of anti-apoptotic proteins could be correlated with the ability of GAC 17:1 to induce apoptosis in multiple myeloma cells. We further found that GAC 17:1 treatment increases the sub-G1 DNA content in a dose-dependent manner. This result indicated that the inhibition of Cyclin D1 expression by GAC 17:1 may be associated with its ability to induce accumulation of the cells in sub-G1 phase [54]. We also observed that Annexin V positive cells are clearly increased in GAC 17:1-treated cells in a dose-dependent manner. GAC 17:1-induced apoptosis was further confirmed by TUNEL assay, Live/Dead assay and loss of mitochondrial membrane potential. We also noted that GAC 17:1 treatment can activate caspase-3 and induce PARP cleavage in a dose-dependent manner. Overall, our results indicate for the first time that GAC 17:1 can suppress constitutive and inducible STAT3 signaling pathway, inhibit growth, and induce substantial apoptosis through the up-regulation of PTPs. This presents a rationale for the use of GAC 17:1 as an anticancer agent to improve the existing therapeutic options for multiple myeloma in near future. Further studies in animals may provide further insight into the potential application of GAC 17:1 in the therapy of multiple myeloma and other cancers.

## 4. Materials and Methods

### 4.1. Reagents

Ginkgolic acid C 17:1 (GAC 17:1) (95% purity) and ginkgolic acid C 15:1 (GAC 15:1) (98% purity), anti-STAT3, anti-SHP-1, anti-PTEN, anti-Bcl-2, anti-Bcl-xL, anti-survivin, anti-IAP-1, anti-COX-2, anti-MMP-9, anti-MMP-2, anti-caspase-3, anti-PARP, anti-β-actin, and horseradish peroxidase (HRP)-conjugated secondary antibodies were purchased from Santa Cruz Biotechnology (Santa Cruz, CA, USA). 3-(4,5-dimethylthiazol-2-yl)-2,5-diphenyltetrazolium bromide (MTT), Tris base, glycine, NaCl, sodium dodecylsulfate (SDS), and bovine serum albumin (BSA) were purchased from Sigma-Aldrich (St. Louis, MO, USA). RPMI 1640, MEM, and fetal bovine serum (FBS) were obtained from Thermo Fisher Scientific Inc. (Waltham, MA, USA). Annexin V was obtained from BD Biosciences (Palo Alto, CA, USA). Anti-p-STAT3 (Tyr705), anti-p-JAK1 (Tyr1022/1023), anti-JAK1, anti-p-src (Tyr416), anti-src, anti-cyclin D1, and anti-cleaved caspase-3 were purchased from Cell Signaling Technology (Beverly, MA, USA). pMXs-gw (#18656) and pMXs-STAT3C (#13373) were obtained from Addgene (Cambridge, MA, USA).

### 4.2. Cell Lines

U266, MM.1S and MEF cells were purchased from American Type Culture Collection (Manassas, VA, USA). U266 and MM.1S cells were cultured in RPMI1640 medium containing 10% FBS, penicillin (100 units/mL), and streptomycin (100 μg/mL). MEF cells were incubated with DMEM medium containing 15% FBS, penicillin (100 units/mL), and streptomycin (100 μg/mL).

### 4.3. Isolation of Human Peripheral Blood Mononuclear Cells (PBMCs)

Human peripheral blood mononuclear cells (PBMCs) were isolated from the blood of healthy adult volunteer donors by density gradient centrifugation on Lymphoprep (Axis-Shield PoC AS, Oslo, Norway).

### 4.4. Western Blot Analysis

Western blot analysis was performed as previous described [59]. Briefly cells were washed with 1× PBS and lysed in 1× cell lysis buffer. 50 μg of the whole cell extract was taken for western blot analysis. First, the protein was resolved in SDS page gel and transferred to nitrocellulose membrane. The membranes were then blocked in blocking buffer to reduce background noise, and probed for protein of interest using target specific primary antibody overnight at 4°. The membranes were washed with 0.1% TBST, and incubated for 1 h with horse radish peroxidase (HRP) tagged secondary antibody. The blots were then examined for the presence or absence of target protein using chemiluminescence substrate (Millipore, Bedford, MA, USA).

### 4.5. Immunocytochemistry for STAT3 Localization

Cells were attached by centrifuging and fixed in 4% paraformaldehyde (PFA) for 20 min at room temperature following the treatment of GAC 17:1 for 3 h, the cells were then permeabilised using 0.2% triton X-100 and blocked with 5% BSA for 1 h. Next, the cells were incubated with human STAT3 or phospho-STAT3 antibody overnight at 4 °C. The slides were then washed with PBS and incubated with FITC-tagged secondary antibody (Jackson ImmunoResearch, West Grove, PA, USA) for 1 h at room temperature, washed with PBS and stained with 1 μg/mL DAPI solution. The slides were then taken for imaging. The cells were excited for DAPI stain at 405 nm and FITC fluorescence at 488 nm and the emitted images signals were captured using a FluoView FV1000 confocal microscope (Olympus, Tokyo, Japan).

### 4.6. EMSA for STAT3-DNA Binding

STAT3-DNA binding was analyzed by EMSA using a 5′-biotinylated STAT3 oligonucleotide (5′-GATCCTTCTGGGAATTCCTAG ATC-3′ and 3′-CTAGGAAGACCCTTAAGGATCTAG-5′) as described in [53]. GAC 17:1-treated cells were collected and nuclear extract was prepared following standard protocol. The nuclear extract protein was then incubated with 5′-biotinylated STAT3 oligonucleotide probe. The protein-oligonucleotide complex was then separated on a 5% native polyacrylamide gel and transferred to nylon membrane and detected using LightShift^®^ Chemiluminescent EMSA kit following the manufacturer’s instructions (Thermo Fisher Scientific Inc.).

### 4.7. RNA Analysis and Reverse Transcription Polymerase Chain Reaction

Total RNA was extracted using a Trizol reagent according to the manufacturer’s instructions (Invitrogen, Carlsbad, CA, USA).The following pairs of forward and reverse primer sets were used PTEN (5′-TTTCTAACCGTGCAGCCTCTT-3′ and 5′-AGCTGTGGTGGGTTATGGTCT-3′) and SHP-1 (5′-GGCTTCTGGGAGGAGTTTGAG-3′ and 5′-CGGAGTTTGTATTCGGTTGTG-3′). All conditions of RT-PCR was performed as described previously [59].

### 4.8. Electroporation-Mediated Transfection in U266 and MEF Cells

Briefly, one million U266 cells were suspended in 120 μL of Neon resuspension buffer R and added 50 nM of PTEN, SHP-1 or scrambled siRNA and transfected using the Neon™ Transfection System (Invitrogen, Carlsbad, CA, USA) following manufacturer instructions. U266 cells were pulsed twice with a voltage of 1150. Similarly, MEF melanoma cells were transfected with 2 μg of pMXs-STAT3C, a dominant active mutant which could express consistently activated STAT3 or pMXs-gw, control vector, in the same manner as described above. After 24 h of transfection, the cells were treated with GAC 17:1 (50 μM) and whole-cell extracts were prepared for Western blotting.

### 4.9. Cell Cycle Analysis

Apoptotic cells were determined by fluorescence-activated cell sorting (FACS) analysis with propidium iodide (PI). U266 cells (1 × 10^6^) were treated with GAC 17:1 for 24 h, washed with PBS, and fixed with 70% ethanol. After fixation, the cells were then washed and incubated in PBS containing 0.1% RNase A at 37 °C for 30 min and then stained with propidium iodide. The apoptotic cells were then analyzed using a using a flow cytometer (Becton-Dickinson, Heidelberg, Germany).

### 4.10. Annexin V assay

To determine if the cells are undergoing early phases of apoptosis before the loss of cell membrane integrity and permits measurements of the kinetics of apoptotic death in relation to the cell cycle. U266 cells (1 × 10^6^) were treated with GAC 17:1 for 24 h, washed with PBS and stained with FITC tagged Annexin V antibody and propidium iodide and analyzed using a flow cytometer (Becton-Dickinson).

### 4.11. TdT-Mediated dUTP Nick end Labeling (TUNEL) Assay

TUNEL assay was performed using a TUNEL assay kit according to manufacturer’s instructions (Roche, Mannheim, Germany). Briefly, U266 cells were treated with GAC 17:1 (50 μM) for 24 h, washed and the cells were fixed with 4% paraformaldehyde for 30 min at RT. The cells were then resuspended in TUNEL reaction solution and incubated for 1 h in dark. The TUNEL positive cells were analyzed by flow cytometer using a FACScan (Becton-Dickinson).

### 4.12. Mitochondrial Membrane Potential

Flow cytometric analysis was performed to determine mitochondrial membrane potential. U266 cells were treated with GAC 17:1 (50 μM) for 24 h, cells were collected, washed with 1× PBS and treated with 5 μM TMRE (tetramethylrhodamine, ethyl ester) for 30 min. The reduction in mitochondrial membrane potential was determined using a flow cytometer (Becton-Dickinson). Acquisition and analysis of the data were performed by using Cell Quest 3.0 software (BD Biosciences, San Jose, CA, USA).

### 4.13. Live/Dead Assay

To assess cytotoxicity, we used a Live/Dead assay kit (Invitrogen) as described in [60].

### 4.14. MTT Assay

The cell viability was measured by using MTT assay. Briefly, U266 and MM.1S melanoma cells were incubated with different concentration of GAC 17:1. At the end of treatment period, 30 μL of MTT solution (2 mg/nL) was added directly to the cells and incubated for an additional 2 h. The formed formazan crystals were then dissolved in DMSO and the absorbance was measured at 570 nm using an automated spectrophotometric plate reader. Cell viability was normalized as relative percentages in comparison with untreated controls.

### 4.15. Real-Time Quantitative PCR

To determine the expression levels of Bcl-2, Bcl-xL, and Cyclin D1 real-time quantitative PCR was performed as described in the manufacturer’s protocol (Applied Biosystems, Waltham, MA, USA). 2^−ΔΔ*C*t^ value was determined with StepOne software (Applied Biosystems). Briefly, total RNA was extracted using Trizol reagent following manufacturer’s instructions (Invitrogen, Life Technologies). 1 μg total RNA was converted to cDNA by M-MLV reverse transcriptase (Promega, Fitchburg, WI, USA). The following pairs of forward and reverse primer sets were used Bcl-2 (5′-TCCCTCGCTGCACAAATACTC-3′ and 5′-GACGACCCGATGGCCATA-3′), Bcl-xL (5′-TACCAGCCTGACCAATATGGC-3′ and 5′-TGGGTTCAAGTGATTCTCCTG-3′), and Cyclin D1, (5′-AGAAGCTGTGCATCTACACCGACA-3′ and 5′-AGAAGCTGTGCATCTACACCGACA-3′). GAPDH (5′-ACCTGACCTGCCGTCTAGAAAA-3′ and 5′-ACGCCTGCTTCACCACCTT-3′) was used as a house keeping gene and endogenous control.

### 4.16. Statistical Analysis

Statistical significance of the data was determined using the Student unpaired *t*-test. All numeric values are represented as the mean ± SD. Significance was set at *p* < 0.05.
